# Pathophysiology, Treatment, and Prognosis of Thrombocytopenia, Anasarca, Fever, Reticulin Fibrosis/Renal Failure, and Organomegaly (TAFRO) Syndrome: A Review

**DOI:** 10.3390/cimb46100668

**Published:** 2024-10-09

**Authors:** Takuya Kakutani, Riko Kamada, Yotaro Tamai

**Affiliations:** 1 Division of Rheumatology, Shonan Kamakura General Hospital, Kanagawa 247-8533, Japan; 2 Division of Hematology, Shonan Kamakura General Hospital, Kanagawa 247-8533, Japan

**Keywords:** TAFRO syndrome, type 1 interferon, IL-6, PI3K/Akt/mTOR, JAK-STAT

## Abstract

TAFRO syndrome, first reported in 2010, is a systemic inflammatory disease with a rapid onset and potentially fatal course if not treated promptly and appropriately. The name is derived from the initial letters describing the characteristic symptoms of thrombocytopenia, anasarca, fever, reticulin fibrosis/renal failure, and organomegaly. It is sometimes considered a special subtype of idiopathic multicentric Castleman disease (iMCD) because lymph node biopsies often reveal the pathology findings seen in iMCD. However, its clinical manifestations and prognoses are not well documented. Since the clinical manifestations and prognoses of TAFRO syndrome differ significantly from those of iMCD, it is recognized as an independent disease concept and considered to partially overlap with the pathology of MCD. The pathogenesis of TAFRO syndrome remains largely unknown. Due to the lack of appropriate treatment, it often presents with multiple organ dysfunction and fatality. In this review, we summarized new findings on the pathogenesis of TAFRO syndrome and discussed current effective therapies and future treatment strategies.

## 1. Introduction

**Figure 1:**
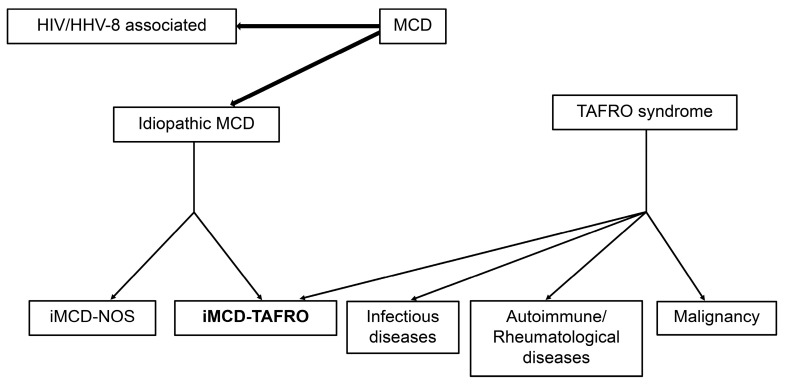
Classification of MCD and TAFRO syndrome. iMCD-TAFRO syndrome is classified as a combination of cases that present with symptoms similar to TAFRO syndrome and have histological evaluations that reveal MCD-like findings and cases that do not undergo histological evaluations and exclude other diseases similar to TAFRO syndrome.

In 2010, Takai et al. reported three cases of thrombocytopenia with mild fibrosis of the bone marrow in association with fever, pleural ascites, and hepatosplenomegaly [1]; based on these symptoms, the acronym TAFRO syndrome (representing thrombocytopenia, anasarca, fever, reticulin fibrosis/renal failure, and organomegaly) was proposed as a new disease. Initially, most cases were reported in Japan; however, the number of cases reported worldwide has increased [2,3,4,5,6,7,8,9]. However, an epidemiological study conducted in Ishikawa Prefecture, Japan, estimated the number of cases to be 110–502 per year (0.9–4.9 per 1 million population), with an estimated prevalence of 860, making it still a rare disease [10]. TAFRO syndrome has a few histopathological features similar to multicentric Castleman disease (MCD) and was considered a subtype of MCD. However, MCD is a systemic inflammatory disease that often has a relatively chronic course with enlarged lymph nodes. Thus, idiopathic MCD (iMCD) and TAFRO syndromes were considered different conditions. Therefore, among the histologically diagnosed iMCD cases, those with a clinical course similar to that of TAFRO syndrome were classified as iMCD–TAFRO, and the others were classified as iMCD not otherwise specified (NOS). Further, patients with a rapid clinical course that could not be biopsied, or with small lymph nodes that were difficult to biopsy and for which no tissue evaluation could be conducted, were proposed to be classified as having TAFRO syndrome after infectious diseases, malignancies, and autoimmune diseases that exhibit symptoms similar to those of the TAFRO syndrome had been excluded from the differential (Figure 1) [11].

The evidence for treatment is limited, and the choice of treatment is based on previous case reports and the course of cases registered in retrospective studies. In most cases, high-dose glucocorticoids are the first choice. However, <10% of patients achieve remission with a single drug [12]. Therefore, tocilizumab, rituximab, and cyclosporine are chosen as second-line therapies in most cases, but their efficacy in iMCD–TAFRO syndrome has not yet been established [13]. This study reviews the classification and mechanisms of TAFRO syndrome in line with new evidence and summarizes the treatment targets.

## 2. Classification Criteria for TAFRO Syndrome

**Figure 2:**
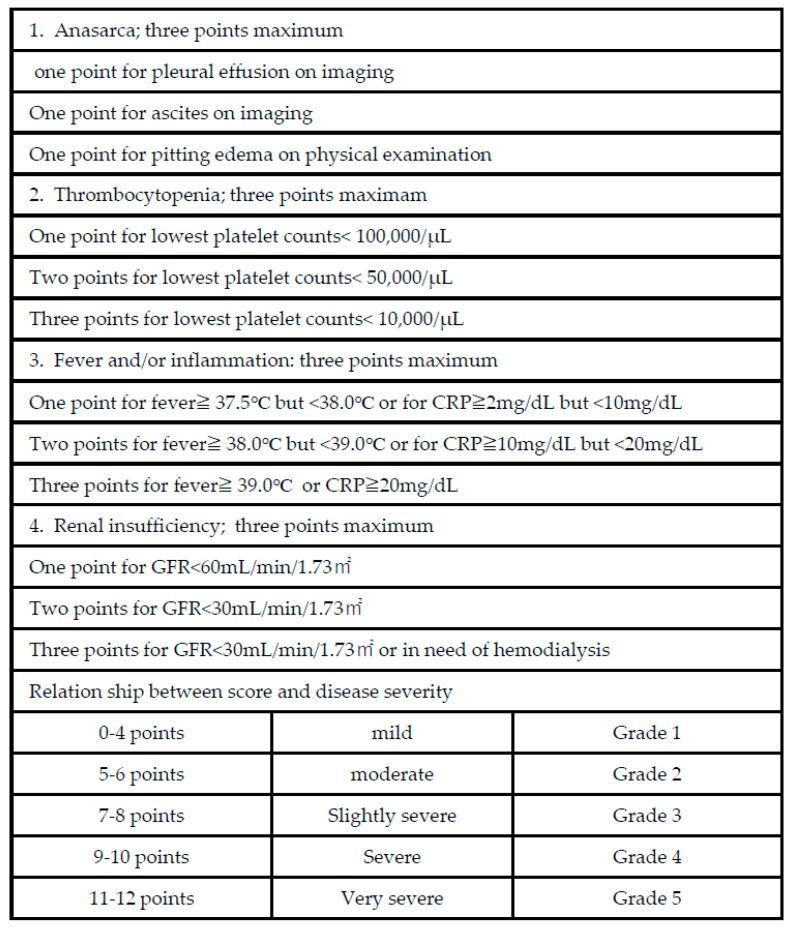
The 2015 disease severity classification for TAFRO syndrome. Minor update added in 2019.

**Figure 3:**
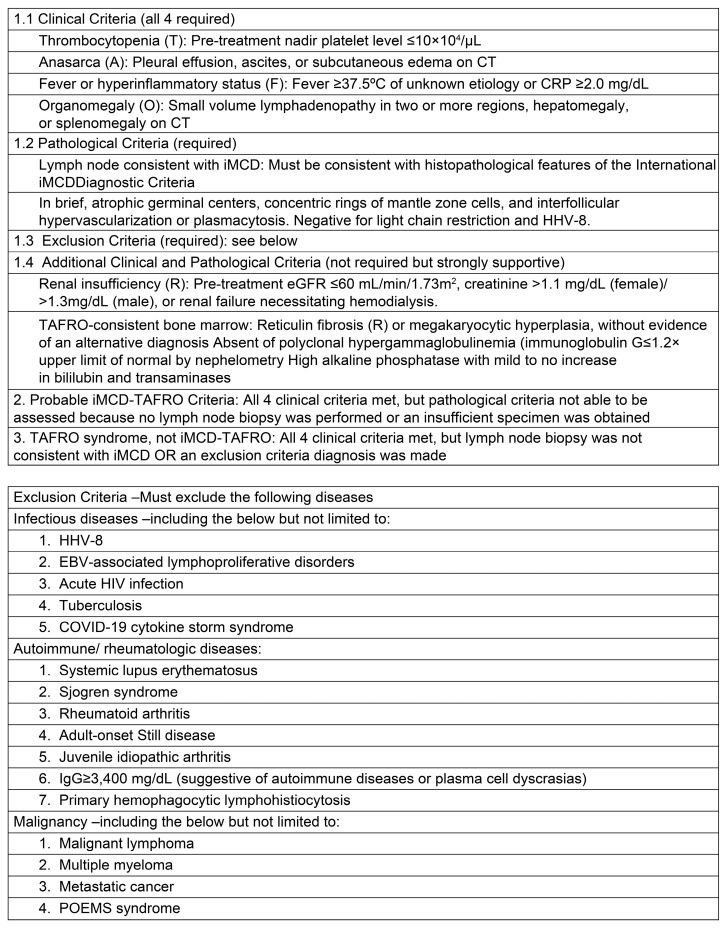
Diagnostic criteria for TAFRO syndrome created in 2021.

Masaki et al. published the first diagnostic and severity criteria for TAFRO syndrome in 2015 [14]. It was developed based on data from 28 patients without accurate diagnoses, resulting in a significant selection bias. It was subsequently updated in 2019 with a few modifications (Figure 2) [11]. Although the evidence regarding iMCD and TAFRO syndromes has improved, TAFRO syndrome and iMCD–TAFRO are not clearly distinguished. The international definition of iMCD–TAFRO was published in 2021 (Figure 3) [15].

**Figure 4:**
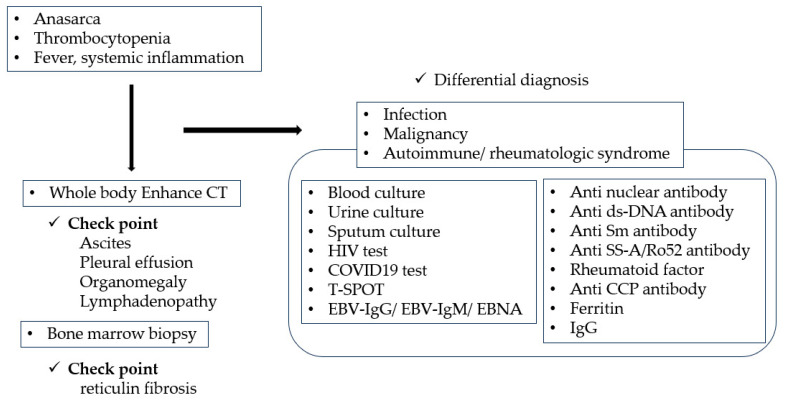
Diagnostic algorithm for TAFRO syndrome.

Distinguishing TAFRO syndrome from other disorders that resemble TAFRO syndrome is important. Although tissue evaluation is desirable whenever possible, the characteristic organ and lymph node enlargements are less noticeable due to the rapid progression of TAFRO syndrome; therefore, therapeutic intervention should not be delayed by focusing extensively on tissue evaluation. The diagnostic algorithms for TAFRO syndrome based on symptoms are shown in Figure 4. It is important to exclude infection, malignant tumor, and lymphoproliferative disease as differential diagnoses. In addition to lymph node evaluation, bone marrow evaluation should also be considered, if possible.

### MCD

MCD was first described in 1956 by the pathologist Castleman B, who reported an anterior mediastinal tumor with characteristic histopathological findings [16]. The lymphoproliferative disease characterized by lymph follicular hyperplasia and hyaline vessels penetrating the germinal center was named Castleman disease and was histopathologically classified as a hyaline-vascular type (HV), a plasma-cell type (PC), in which the plasma cells proliferated in sheets in the interfollicular region of the lymph nodes; and a mixed type, in which a mixture of the two types was observed. It was also classified as monogenic and polycyclic based on the distribution of lesions.

In Europe and the United States, most cases are related to human immunodeficiency virus (HIV) infection and positive human herpesvirus-8 (HHV-8), which virally expresses interleukin (IL)-6. Platelet hyperplasia occurs in the bone marrow during accelerated megakaryocyte differentiation [17]. iMCD has an increased platelet count, reflecting inflammation. HIV-negative and HHV-8-negative cases also exist and are common in Japan. Moreover, in the United States, more than half of the cases are HHV-8-negative MCD, according to a report by Fajgenbaum et al. in 2014, who named it iMCD [18].

Additionally, a recent report from Japan suggested that anti-SS-A/Ro60 antibodies may be involved in the disease characteristics of TAFRO syndrome, which may contribute to understanding the disease and selecting treatment methods in the future [19].

## 3. Differences between TAFRO Syndrome and iMCD

TAFRO syndrome is a rapid-onset systemic inflammatory disease that can lead to multiple organ failure and fatality without early and appropriate therapeutic intervention. The syndrome has been reported in all age groups ranging from childhood to older adults, and the condition shows a male predilection. The initial symptoms include fever and general malaise, followed by a rapid onset of generalized edema, thrombocytopenia, and renal dysfunction. The laboratory findings are characterized by a marked increase in alkaline phosphatase, without an increase in lactate dehydrogenase, and normal-to-low levels of γ-globulin, in contrast to the findings in iMCD [20]. The immature platelet fraction (IPF) and mean platelet volume (MPV) are elevated, and thrombocytopenia is often maintained initially and subsequently declines rapidly. Careful judgment is needed to avoid dismissing platelets because they are well maintained. In iMCD, megakaryocytes are stimulated by cytokines released with the involvement of IL-6, resulting in thrombocytosis. Conversely, TAFRO syndrome is an inflammatory condition that causes fibrosis in the bone marrow for unclear reasons, resulting in abnormalities in the bone marrow. It is thought that hematopoiesis is impaired, leading to a decrease in platelets. Some investigators consider it a part of iMCD because of the similarities in histological findings shared between TAFRO and iMCD; however, the clinical presentation is different, and the course is more rapid in TAFRO syndrome. Therefore, we defined iMCD-NOS as iMCD without signs of TAFRO syndrome, iMCD–TAFRO as iMCD with histopathological evidence of iMCD and clinical signs of TAFRO syndrome, and TAFRO syndrome as iMCD with clinical signs of TAFRO syndrome but no histological evidence of TAFRO syndrome [21]. Masaki et al. defined TAFRO cases without iMCD as cases with clinical signs of TAFRO syndrome but no histological findings and reported that iMCD–TAFRO and TAFRO without iMCD belong to the same disease group, which is expected to lead to further disease classification in the future [22].

## 4. Histopathological Findings Regarding TAFRO Syndrome

It is recommended that a lymph node biopsy should be performed whenever possible when TAFRO syndrome is suspected. It is important to exclude mimickers by differentiating lymphoproliferative disorders, such as malignant lymphoma. Kurose et al. compared the pathological findings of lymph nodes in iMCD with and without symptoms of TAFRO syndrome and found that iMCD with TAFRO syndrome was predominantly characterized by atrophy of lymphoid follicles, long interfollicular distances, and increased glomerular vascular proliferation within germinal centers. Patients with characteristics of the hyper-vascular subtype are more likely to secrete VEGF and are therefore more likely to be characteristic of TAFRO syndrome [23]. In addition, plasma cell proliferation is rare in TAFRO syndrome, and when plasma cell proliferation is observed in a patient suspected to have TAFRO syndrome, it is important to consider and differentiate it from other diseases [24].

## 5. Pathophysiology of TAFRO Syndrome

### 5.1. Involvement of Type 1 Interferon

We showed that interferon (IFN)-β stimulation of monocytes and T cells from iMCD–TAFRO samples induces an increased mammalian target for rapamycin (mTOR) activation, and we identified type 1 IFN responses as a common gene signature that is upregulated during iMCD–TAFRO relapse based on other studies. We demonstrated a positive correlation between IFN-1-responsive genes and mTOR gene signatures in classical monocytes and observed increased mTOR activation upon IFN-1 stimulation in vitro. We further showed that this pathway can be inhibited by either mTOR complex 1 (mTORC1) or Janus kinase (JAK) 1/2 inhibition and clarified the mechanism by which IFN-1 signaling contributes to the pathogenesis of iMCD–TAFRO through increased JAK-dependent mTOR activation [25]. Future studies should investigate whether mTOR activation is due to an overreaction to IFN-1 or whether mTOR is activated as a result of elevated IFN-1 levels in the circulation or tissues.

Inhibitors of the Phosphoinositide 3-kinase/protein kinase B/mTOR (PI3K/Akt/mTOR) pathway and the JAK/signal transducers and activators of the transcription (JAK/STAT) pathway have been shown to be effective in iMCD–TAFRO because they bind to the Interferon Alpha and Beta Receptor Submit 1/2 (IFNAR1/2)-receptor complex and activate these pathways through signaling when stimulated by IFN-β. The possibility that the inhibitors of these pathways may be effective has been demonstrated previously [26].

#### 5.1.1. IL-6 Pathways

The binding of IL-6 to membrane-bound IL-6R (mIL-6R) induces the formation of a homodimer of gp130, forming a high-affinity receptor complex composed of IL-6, IL-6R, and gp130. IL-6 production was analyzed by in situ hybridization and immunohistochemistry [27]. This receptor signaling system is referred to as the classical signaling system. Soluble IL-6R (sIL-6R), which lacks the cytoplasmic portion of mIL-6R and is generated by enzymatic cleavage or selective splicing of mIL-6R, also has the ability to bind to IL-6. Subsequently, the IL-6/sIL-6 complex may form a complex with gp130. The formation of IL-6 classical or trans-signaling ligand–receptor complexes lead to the activation of multiple intracellular signaling pathways, including the JAK/STAT pathway, the Ras–Mitogen-activated protein kinase (MAPK) pathway, the p38 and JNKMAPK pathways, and the PI3K/Akt pathway [28].

Although elevated IL-6 levels have been found in the germinal centers of lymph nodes of patients with iMCD, indicating that IL-6 plays an important role in disease pathogenesis [29], the mechanisms underlying IL-6 overproduction in iMCD are not fully understood. A previous study reported a correlation between disease status and IL-6 levels, which can be used as an index of disease activity, suggesting that IL-6 levels may be involved in pathological conditions [30]. Immunohistology in the lymph nodes of patients with iMCD and chemical analysis indicates that IL-6 is produced in the germinal centers of hyperplastic lymph nodes, and B cells and follicular dendritic cells in the germinal centers are the main sources of IL-6 [31].

IL-1β and tumor necrosis factor (TNF)-α are proinflammatory cytokines that stimulate IL-6 production via the nuclear factor kappa-light-chain-enhancer of activated B cells’ (NF-κB) signaling, and elevated levels of these cytokines have been reported in patients with iMCD. In a study examining cytokine profiles, serum IL-10, IL-23, and vascular endothelial growth factor (VEGF)-A levels were elevated in patients with iMCD–TAFRO [32]. In another study, cytokines and chemokines, such as C-X-C motif ligand 13 (CXCL13) and C-C motif chemokine ligand 23 (CCL23), were shown to be strongly involved in iMCD, suggesting that iMCD may be caused by a chemokine storm [33]. In iMCD-NOS and iMCD–TAFRO, CXCL13 was the most abundant cytokine, with expression centered in the lymph node’s germinal centers. Each cytokine has been shown to be associated with clinical symptoms: IL-6 is associated with fever, fatigue, and elevated C-reactive protein (CRP), VEGF-A is associated with fluid retention due to increased vascular permeability, and cytokines, such as CXCL13, may be associated with enlarged lymph nodes in iMCD-NOS. iMCD–TAFRO may have different profiles of cytokines and chemokines, and thus may present with specific symptoms [26]. Further studies are required to accumulate additional cases.

#### 5.1.2. PI3K/Akt/mTOR Pathway

A previous study reported that sirolimus, an mTOR inhibitor, was effective in patients with recurrent MCD refractory to IL-6 inhibitors; flow cytometry showed activation of CD8+ T cells and elevated VEGF-A levels, and proteomic analysis showed increased activity of the PI3K/Akt/mTOR pathway [34]. Patients treated with sirolimus achieved clinical remission with decreased CD8+ T cell and VEGF-A levels. In another report, in a case of IL-6 inhibitor relapse, sirolimus-treated patients were evaluated by RNA sequencing of peripheral blood CD4+ T cells before and after treatment. The results showed that treatment with sirolimus reduced mTOR-related pathways, such as mTOR, Huntington, eIF4, and p70S6K signaling. This suggests the existence of a pathway different from the IL-6 pathway and that mTOR inhibitors, such as sirolimus, may be effective in such cases [35].

#### 5.1.3. mTOR2 Pathway Activation of iMCD–TAFRO

mTOR is a kinase that integrates inputs from various cytokines, growth factors, and other ligands with signals via mTORC1 and mTOR complex 2 (mTORC2) [36]. Sirolimus, a TOR inhibitor, predominantly inhibits mTORC1. Reports on the efficacy of sirolimus in patients with iMCD–TAFRO have been published, with phase-3 clinical trials still ongoing [37]. However, not all cases show a response, and the ACCELERATE registry showed efficacy in 5/11 cases, which was considered to be due to the limited mTORC2 inhibitory effect of sirolimus [38].

To investigate mTORC2 activation in iMCD, pNDRG1, an mTORC2 effector protein, was quantified by immunostaining lymph node tissues from six patients with iMCD–TAFRO and eight patients with iMCD-NOS who did not meet the diagnostic criteria for TAFRO syndrome. Compared to the target tissues, mTORC2 activation was observed in all regions of the lymph nodes in iMCD–TAFRO and in the interfollicular space in iMCD-NOS. We also found increased pNDRG1 expression in the germinal centers of iMCD-TAFRO compared to that in autoimmune lymphoproliferative syndrome (ALPS), an mTOR-driven sirolimus-responsive lymphoproliferative disease [35]. These results suggest that not only increased mTORC2 activity and mTORC1 inhibition in iMCD but also dual mTORC1/mTORC2 inhibition may be a rational therapeutic approach. The role of mTORC2 in the pathogenesis of iMCD remains unclear; although its biological functions are largely unknown, it is thought to regulate cell survival, proliferation, and cytoskeletal pathways.

When activated by PI3K-dependent potentiation factors, mTORC2 phosphorylates and activates Akt and SGK1, followed by the phosphorylation of the cytosolic protein N-Myc Downstream Regulated 1 (NDRG1) to pNDRG1. Thus, pNDRG1 expression can be used as a reliable biomarker of mTORC2 activation [39]. The involvement of mTORC2 in promoting Akt-mediated cell survival and anti-apoptotic signaling in malignant tumors suggests a potential role of mTORC2 in lymphoproliferative diseases, such as iMCD [40].

Sumiyoshi et al. reported increased mTORC2 activation in iMCD–TAFRO and iMCD-NOS compared to healthy lymph nodes and increased mTORC activity in iMCD–TAFRO compared to ALPS. Considering that mTORC2 may be the driver of iMCD pathogenesis and that sirolimus has limited mTORC2 inhibition, mTORC1/mTORC2 inhibition or the JAK/STAT3 pathway upstream of mTOR in iMCD–TAFRO may be effective. However, a previous report showed that sirolimus was effective in three patients with IL-6 inhibitor-refractory iMCD–TAFRO, suggesting that mTORC2 is not the only factor that plays a role in the pathogenesis [34]. Further studies are needed to identify cell types that show increased mTORC2 activity, elucidate its pathogenesis, and discover therapeutic approaches for iMCD–TAFRO.

#### 5.1.4. JAK/STAT3 Pathway

A study of iMCD cases that assessed whether IL-6 inhibitor administration was effective revealed that IL6-JAK-STAT3 signaling was activated in ineffective cases [41]. Furthermore, peripheral blood mononuclear cells from patients with iMCD in remission showed a hypersensitive response to IL-6 stimulation in vitro, indicating that this response can be controlled by JAK1/2 inhibitors [42]. Pierson et al. investigated therapeutic targets in iMCD and used gene set enrichment analysis (GSEA) to evaluate disease factors in siltuximab-non-responsive patients and found that IL-6-JAK-STAT3 signal pathways, NF-κB-mediated TNF-α signal pathways, and allograft rejection were predominant. Immunohistochemistry for pSTAT3, an indicator of JAK-STAT3 activation, was performed to confirm pathway activation at the primary site of iMCD pathology. pSTAT3 expression was predominantly increased in the interfollicular space of iMCD tissues compared to normal tissues, suggesting that pSTAT3 expression is increased in the lymphoid tissue of iMCD. This result indicates that pSTAT3 expression is increased in iMCD lymphoid tissues and that the JAK-STAT3 signaling pathway is activated in iMCD tissues. The enrichment of IL6-JAK-STAT3 signaling pathways in the iMCD serum proteome, the ability of JAK1/2 inhibition to suppress hypersensitivity to cytokine stimulation, increased pSTAT3 expression in iMCD, and the absence of differences in IL-6 and pSTAT3 expression in siltuximab responders and non-responders suggest that the underlying mechanism in the treatment of non-responders, including siltuximab-non-responders, may be regulated by activating ligands other than IL-6 or that JAK/STAT3 pathways may be involved in iMCD development, possibly through aberrant pathways downstream of IL-6. Further studies are needed to determine the mechanism of increased pSTAT3 activation. However, the JAK1/2 inhibitor luxoltinib may be an effective treatment option. Luxoltinib has demonstrated activity in other hyperinflammatory cytokine-driven diseases, such as hemophagocytic lymphohistiocytosis, by suppressing proinflammatory cytokines and reducing T cell proliferation by blocking STAT transmission. Although the use of JAK inhibitors has expanded to various diseases, they are still widely used for the treatment of rheumatoid arthritis. Kadoba et al. reported a case of TAFRO syndrome-like symptoms complicated by rheumatoid arthritis and reported that JAK inhibitors were effective in improving TAFRO symptoms. Their effectiveness has also been reported in clinical practice [43].

## 6. Treatment of TAFRO Syndrome

There are no standard treatment recommendations for the treatment of TAFRO syndrome due to its infrequency and largely unknown etiology, although thalidomide and other drugs have been utilized [44,45,46]. Recent studies have reported the efficacy of luxolutinib in JAK-I [47,48].

### 6.1. Corticosteroids

Early treatment with glucocorticoid pulse therapy is considered to be the key to improving prognoses, and a regimen of methylprednisolone 500–1000 mg/day for 3 days followed by prednisolone 1 mg/kg/day for 2 weeks is recommended as an acute therapy. Rapid therapeutic intervention is extremely important because of the rapid progression of the disease; however, the response rate to glucocorticoid monotherapy is <10% [12].

### 6.2. Anti-IL6 Therapy

There are two anti-IL6 therapies: siltuximab, a human–mouse chimeric monoclonal antibody, and tocilizumab, an anti-IL-6 antibody. Siltuximab was approved for the treatment of iMCD only in North America and Europe after a phase-3 clinical trial showed its efficacy and long-term tolerability compared to supportive care in 2014 [49].

Tocilizumab is a humanized IL-6 antagonist that blocks IL-6 transmembrane signaling and has been approved in Japan for the treatment of MCD. It is recognized as a first-line treatment in combination with glucocorticoids; however, relapse occurs in approximately 66% of patients [50].

### 6.3. mTOR Inhibitor

In iMCD, the mTOR, which functions downstream of the IL-6 signaling pathway, is a kinase that integrates with mTORC1/2 and is considered to be involved in its pathogenesis. The inhibitor sirolimus predominantly suppresses mTORC1 and has been reported to be effective against iMCD. In Japan, a phase-3 study of iMCD is currently underway and is expected to be effective [36]. Efficacy for TAFRO syndrome has not been reported because it is a rare disease, and efficacy reports for iMCD are limited; therefore, it is necessary to examine the efficacy of JAK-1/2 inhibitors in various disease groups.

### 6.4. JAK-1/2 Inhibitor

The JAK/STAT3 pathway plays an important role in the pathogenesis of inflammation in iMCD [41,42]. Therefore, the possibility that luxolutinib, a JAK1/2 inhibitor, may be effective in the treatment of iMCD has attracted attention, and several case reports on both pediatric and adult patients have been published [47,48,51]. In iMCD, JAK1/2 inhibitors are expected to be effective in the treatment of patients with refractory or relapsed cases of anti-IL-6 antibodies, and a similar JAK inhibitor, tofacitinib, has been reported to be effective [52]. We plan to conduct a clinical trial based on iMCD to demonstrate its efficacy.

### 6.5. Anti-CD20 Therapies

Rituximab is a chimeric monoclonal antibody that binds to CD20 and is often selected as second-line therapy [53]. One case report has shown its efficacy as a treatment option for iMCD [54]. In some cases of TAFRO syndrome, plasma cell infiltration is observed in the interfollicular zone of lymph nodes, and IL-6 stimulates the maturation of B cells and promotes immunoglobulin production from plasma cells. Therefore, the combination of RTX and monoclonal anti-BLyS antibody therapy with belimumab has been reported to be effective for treating recurrent TAFRO syndrome [55]. However, no studies have reported consistent efficacy, and the possibility of partial efficacy and failure to maintain long-term remission should be considered.

### 6.6. Calcineurin Inhibitors

Tacrolimus and cyclosporine A, which are calcineurin inhibitors that target activated T cells and inhibit the secretion of proinflammatory cytokines, such as IL-2, have been reported to be effective in several cases [11]. Previously, Iwaki et al. reported that the C-X-C motif chemokine ligand 10 (CXCL10), also known as interferon γ-induced protein 10, was higher in iMCD-TAFRO patients during relapse compared to iMCD-NOS patients [32], and it is known that CXCL10 plays an important role in bringing activated T cells to the site of inflammation [56]. Furthermore, it has been shown that sIL-2Rα, a marker of T cell activation, is upregulated in iMCD-TAFRO patients [34], suggesting that IL-2 may be involved in the pathogenesis of TAFRO syndrome and that calcineurin inhibitors may be effective.

### 6.7. Cytotoxic Chemotherapy

Cytotoxic chemotherapy using cyclophosphamide, doxorubicin, vincristine, and prednisolone, which are used for diffuse large B cell lymphoma (DLBCL), may be used to treat severe iMCD and is effective in most cases; however, relapse is common, and side effects are significant [57].

Other pathways that may contribute to the disease process in iMCD as potential therapeutic options include TNFα, IFNγ, and IL-1β [58]. TNFα signaling via NFκB is also abundant in iMCD and attractive as a target; TNFα can induce the activation of IL-6, VEGF, and JAK-STAT3 and may drive pSTAT3 by stimulating non-IL-6 ligand production. In rheumatoid arthritis and other conditions, anti-TNFα inhibitors suppress cytokine production and increase hemoglobin, thereby reducing inflammation. Considering the proteomic overlap between a few patients with iMCD and rheumatoid arthritis, anti-TNFα inhibitors may also be effective in iMCD. However, further studies are needed.

### 6.8. Thalidomide

Thalidomide is considered to have multifaceted immunomodulatory effects, inhibiting not only IL-6 and VEGF, but also basic fibroblast growth factor-2 and TNF-α. Therefore, it is thought to be effective against iMCD-TAFRO, in which VEGF is thought to be partially involved in the pathology, and there have been several reports showing its effectiveness in the past [59,60,61]. However, there is only one case reported that is thought to be iMCD-TAFRO, reported by Tatekawa et al., and it is difficult to consider it as a standard treatment due to the complexity of the pathology of iMCD-TAFRO, so further case accumulation is required [62].

## 7. Prognosis

The prognosis for TAFRO syndrome is poor due to the difficulty of diagnosing it, its low recognition, and the lack of established treatments. The 5-year survival rate is reported to be 66.5%. After 24 months of disease progression, the overall survival rate drops sharply, with more than one-third of patients dying [63,64].

## 8. Conclusions—Future Directions

**Figure 5:**
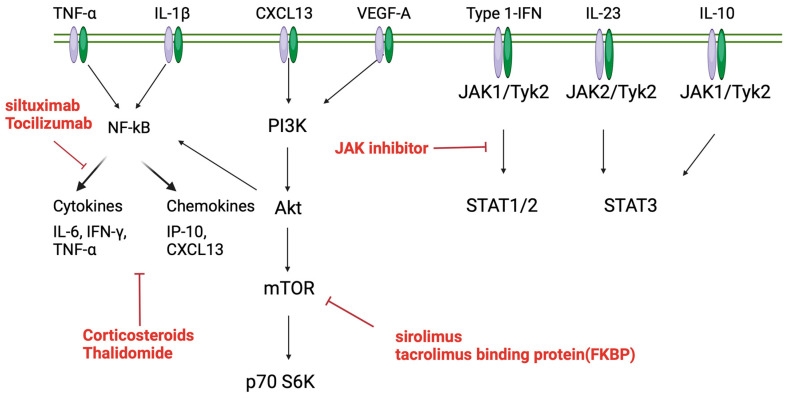
Pathophysiology and potential therapeutic targets in TAFRO syndrome (other than IL-6 pathway).

**Figure 6:**
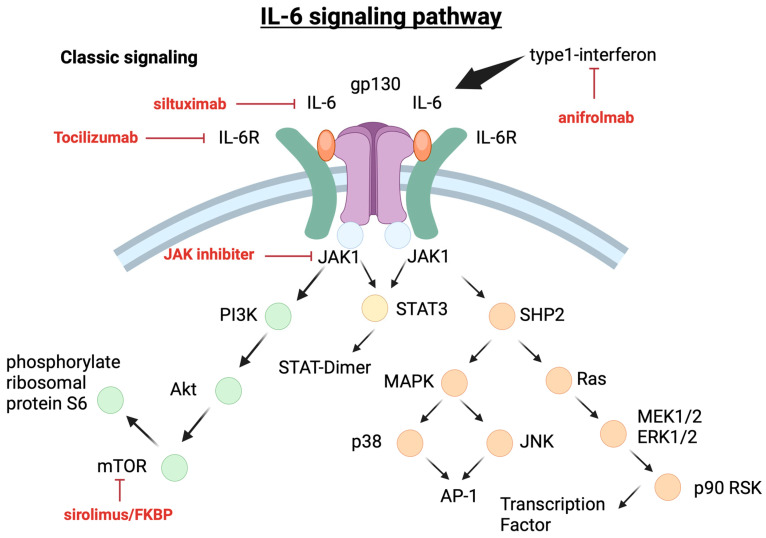
IL-6 signaling pathway and therapeutic targets.

TAFRO syndrome is a rare disease, and this review has described its pathophysiology, classification, and treatment based on basic and clinical research. Figure 5 and Figure 6 show the involvement of cytokines and chemokines in iMCD and their potential activation pathways, as well as corresponding therapeutic agents. Currently, evidence-based therapies for this condition have not been established, and the response rates to standard therapies remain low. The role of anti-IL-6 antibodies has been highlighted as potentially effective, given their targeting of the IL-6 pathway involved in the disease’s pathology. However, high recurrence rates persist. While the central suppression of the IL-6 pathway may seem adequate, this is also applicable to the mTOR pathway, and both mTORC1/2 may be involved in pathological conditions. Therefore, the selection of combination therapy for severe cases is necessary. Furthermore, the cytokines and chemokines involved in iMCD-NOS and iMCD-TAFRO and TAFRO syndrome are different. It is known that iMCD-NOS is mainly characterized by IPL-like plasma cell infiltration and anti-IL-6 antibodies are relatively effective, but it is thought that various factors other than IL-6 are involved in the cytokines and chemokines involved in iMCD-TAFRO and TAFRO syndrome. It is suggested that clarifying their involvement may lead to treatment selection in TAFRO syndrome, which shows various degrees of severity. It is considered important to create an algorithm for selecting treatment according to the severity, and further information regarding the effectiveness of JAK inhibitors, which are considered to be important drugs, is awaited. Currently, clinical trials are being conducted on patients with iMCD-NOS. It would be ideal to verify this involvement in iMCD-TAFRO and TAFRO syndrome as well, but, due to its rarity, this would be difficult, so it would be more realistic to collect case reports, case series, etc. and verify them.

## Data Availability

No new data were created or analyzed in this study. Data sharing is not applicable to this article.

## References

[1] Takai K., Nikkuni K., Shibuya H., Hashidate H. (2010). [Thrombocytopenia with mild bone marrow fibrosis accompanied by fever, pleural effusion, ascites and hepatosplenomegaly]. Rinsho Ketsueki.

[2] Inoue M., Ankou M., Hua J., Iwaki Y., Hagihara M., Ota Y. (2013). Complete resolution of TAFRO syndrome (thrombocytopenia, anasarca, fever, reticulin fibrosis and organomegaly) after immunosuppressive therapies using corticosteroids and cyclosporin A: A case report. J. Clin. Exp. Hematop..

[3] Iwaki N., Sato Y., Takata K., Kondo E., Ohno K., Takeuchi M., Orita Y., Nakao S., Yoshino T. (2013). Atypical hyaline vascular-type Castleman’s disease with thrombocytopenia, anasarca, fever, and systemic lymphadenopathy. J. Clin. Exp. Hematop..

[4] Masaki Y., Nakajima A., Iwao H., Kurose N., Sato T., Nakamura T., Miki M., Sakai T., Kawanami T., Sawaki T. (2013). Japanese variant of multicentric Castleman’s disease associated with serositis and thrombocytopenia—A report of two cases: Is TAFRO syndrome (Castleman- Kojima disease) a distinct clinicopathological entity?. J. Clin. Exp. Hematop..

[5] Louis C., Vijgen S., Samii K., Chalandon Y., Terriou L., Launay D., Fajgenbaum D.C., Seebach J.D., Muller Y.D. (2017). TAFRO syndrome in Caucasians: A case report and review of the literature. Front. Med..

[6] Hiramatsu S., Ohmura K., Tsuji H., Kawabata H., Kitano T., Sogabe A., Hashimoto M., Murakami K., Imura Y., Yukawa N. (2016). Successful treatment by rituximab in a patient with TAFRO syndrome with cardiomyopathy. Nihon Rinsho Meneki Gakkai Kaishi.

[7] José F.F., Kerbauy L.N., Perini G.F., Blumenschein D.I., Pasqualin D.D.C., Malheiros D.M.A.C., Campos Neto G.C., de Souza Santos F.P., Piovesan R., Hamerschlak N. (2017). A life-threatening case of TAFRO syndrome with dramatic response to tocilizumab, rituximab, and pulse steroids: The first case report in Latin America. Medicine.

[8] Morisawa N., Satoh H., Matsuyama M., Hayashi N., Adachi A., Satoh J.I., Yokoo T., Amemiya M. (2016). [Usefulness of the treatment with corticosteroids and ciclosporin A for TAFRO syndrome]. Nihon Naika Gakkai Zasshi.

[9] Suzuki K., Nakamura K., Kasuya T., Yamasaki K., Tokinaga K., Tashiro J., Kimura A., Noro M., Akikusa B., Kojima M. (2016). Case Report A case of TAFRO syndrome successfully treated with intravenous cyclophosphamide therapy. Nihon Naika Gakkai Zasshi.

[10] Masaki Y., Kawabata H., Fujimoto S., Kawano M., Iwaki N., Kotani T., Nakashima A., Kurose N., Takai K., Suzuki R. (2019). Epidemiological analysis of multicentric and unicentric Castleman disease and TAFRO syndrome in Japan. J. Clin. Exp. Hematop..

[11] Fujimoto S., Kawabata H., Sakai T., Yanagisawa H., Nishikori M., Nara K., Ohara S., Tsukamoto N., Kurose N., Yamada S. (2021). Optimal treatments for TAFRO syndrome: A retrospective surveillance study in Japan. Int. J. Hematol..

[12] Wu X., Zhang X., Qian S., Shi C., Li X., Feng X., Zhu L., Ge J., Li Z., Zhang M. (2023). The experience of diagnosis and treatment for TAFRO syndrome. Ann. Hematol..

[13] Masaki Y., Kawabata H., Takai K., Kojima M., Tsukamoto N., Ishigaki Y., Kurose N., Ide M., Murakami J., Nara K. (2016). Proposed diagnostic criteria, disease severity classification and treatment strategy for TAFRO syndrome, 2015 version. Int. J. Hematol..

[14] Masaki Y., Kawabata H., Takai K., Tsukamoto N., Fujimoto S., Ishigaki Y., Kurose N., Miura K., Nakamura S., Aoki S. (2020). 2019 Updated diagnostic criteria and disease severity classification for TAFRO syndrome. Int. J. Hematol..

[15] Nishimura Y., Fajgenbaum D.C., Pierson S.K., Iwaki N., Nishikori A., Kawano M., Nakamura N., Izutsu K., Takeuchi K., Nishimura M.F. (2021). Validated international definition of the thrombocytopenia, anasarca, fever, reticulin fibrosis, renal insufficiency, and organomegaly clinical subtype (TAFRO) of idiopathic multicentric Castleman disease. Am. J. Hematol..

[16] Castleman B., Iverson L., Menendez V.P. (1956). Localized mediastinal lymphnode hyperplasia resembling thymoma. Cancer.

[17] Masaki Y., Ueda Y., Yanagisawa H., Arita K., Sakai T., Yamada K., Mizuta S., Fukushima T., Takai K., Aoki S. (2023). TAFRO syndrome. Nippon Rinsho.

[18] Fajgenbaum D.C., van Rhee F., Nabel C.S. (2014). HHV-8-negative, idiopathic multicentric Castleman disease: Novel insights into biology, pathogenesis, and therapy. Blood.

[19] Shirakashi M., Nishida Y., Nakashima R., Fujimoto M., Hiwa R., Tsuji H., Kitagori K., Akizuki S., Morinobu A., Yoshifuji H. (2024). TAFRO syndrome is associated with anti-SSA/Ro60 antibodies, in contrast to idiopathic Castleman disease. Sci. Rep..

[20] Ide M., Yokoyama T., Ishikawa M., Kojima K. (2023). Stepwise treatment for TAFRO syndrome. J. Med. Cases.

[21] Masaki Y., Arita K., Sakai T., Takai K., Aoki S., Kawabata H. (2022). Castleman disease and TAFRO syndrome. Ann. Hematol..

[22] Fujimoto S., Sakai T., Kawabata H., Kurose N., Yamada S., Takai K., Aoki S., Kuroda J., Ide M., Setoguchi K. (2019). Is TAFRO syndrome a subtype of idiopathic multicentric Castleman disease?. Am. J. Hematol..

[23] Kurose N., Futatsuya C., Mizutani K.I., Kumagai M., Shioya A., Guo X., Aikawa A., Nakada S., Fujimoto S., Kawabata H. (2018). The clinicopathological comparison among nodal cases of idiopathic multicentric Castleman disease with and without TAFRO syndrome. Hum. Pathol..

[24] Nishimura M.F., Nishimura Y., Nishikori A., Yoshino T., Sato Y. (2022). Historical and pathological overview of Castleman disease. J. Clin. Exp. Hematop..

[25] Pai R.L., Japp A.S., Gonzalez M., Rasheed R.F., Okumura M., Arenas D., Pierson S.K., Powers V., Layman A.A.K., Kao C. (2020). Type I IFN response associated with mTOR activation in the TAFRO subtype of idiopathic multicentric Castleman disease. JCI Insight.

[26] Sumiyoshi R., Koga T., Kawakami A. (2022). Candidate biomarkers for idiopathic multicentric Castleman disease. J. Clin. Exp. Hematop..

[27] Kishimoto T., Akira S., Narazaki M., Taga T. (1995). Interleukin-6 family of cytokines and gp130. Blood.

[28] Yoshizaki K., Murayama S., Ito H., Koga T. (2018). The role of interleukin-6 in Castleman disease. Hematol. Oncol. Clin. N. Am..

[29] Leger-Ravet M.B., Peuchmaur M., Devergne O., Audouin J., Raphael M., Van Damme J., Galanaud P., Diebold J., Emilie D. (1991). Interleukin-6 gene expression in Castleman’s disease. Blood.

[30] Suzuki H., Sano T., Shimasaki Y., Yamaguchi M., Ide T., Arinaga-Hino T., Kuwahara R., Amano K., Oshima K., Nagafuji K. (2022). TAFRO syndrome that responded to prednisolone-only treatment: Evaluating changes in IL-6. Intern. Med..

[31] Yoshizaki K., Matsuda T., Nishimoto N., Kuritani T., Taeho L., Aozasa K., Nakahata T., Kawai H., Tagoh H., Komori T. (1989). Pathogenic significance of interleukin-6 (IL-6/BSF-2) in Castleman’s disease. Blood.

[32] Iwaki N., Gion Y., Kondo E., Kawano M., Masunari T., Moro H., Nikkuni K., Takai K., Hagihara M., Hashimoto Y. (2017). Elevated serum interferon γ-induced protein 10 kDa is associated with TAFRO syndrome. Sci. Rep..

[33] Pierson S.K., Stonestrom A.J., Shilling D., Ruth J., Nabel C.S., Singh A., Ren Y., Stone K., Li H., van Rhee F. (2018). Plasma proteomics identifies a ‘chemokine storm’ in idiopathic multicentric Castleman disease. Am. J. Hematol..

[34] Fajgenbaum D.C., Langan R.A., Japp A.S., Partridge H.L., Pierson S.K., Singh A., Arenas D.J., Ruth J.R., Nabel C.S., Stone K. (2019). Identifying and targeting pathogenic PI3K/AKT/mTOR signaling in IL-6-blockade-refractory idiopathic multicentric Castleman disease. J. Clin. Investig..

[35] Sumiyoshi R., Koga T., Furukawa K., Umeda M., Yamamoto K., Mori R., Kawakami A. (2021). A case of tocilizumab-refractory idiopathic multicentric Castleman’s disease successfully treated with sirolimus. Clin. Immunol..

[36] Saxton R.A., Sabatini D.M. (2017). MTOR signaling in growth, metabolism, and disease. Cell.

[37] Koga T., Hagimori N., Takemori S., Morimoto S., Sumiyoshi R., Shimizu T., Hosogaya N., Fukushima C., Yamamoto H., Kawakami A. (2020). Randomized, double-blind, placebo-controlled, parallel-group trial of sirolimus for tocilizumab-resistant idiopathic multicentric Castleman disease: Study protocol for clinical trial. Medicine.

[38] Phillips A.D., Kakkis J.J., Tsao P.Y., Pierson S.K., Fajgenbaum D.C. (2022). Increased mTORC2 pathway activation in lymph nodes of iMCD-TAFRO. J. Cell. Mol. Med..

[39] García-Martínez J.M., Alessi D.R. (2008). mTOR complex 2 (mTORC2) controls hydrophobic motif phosphorylation and activation of serum- and glucocorticoid-induced protein kinase 1 (SGK1). Biochem. J..

[40] Kim L.C., Cook R.S., Chen J. (2017). mTORC1 and mTORC2 in cancer and the tumor microenvironment. Oncogene.

[41] Pierson S.K., Shenoy S., Oromendia A.B., Gorzewski A.M., Langan Pai R.A., Nabel C.S., Ruth J.R., Parente S.A.T., Arenas D.J., Guilfoyle M. (2021). Discovery and validation of a novel subgroup and therapeutic target in idiopathic multicentric Castleman disease. Blood Adv..

[42] Arenas D.J., Floess K., Kobrin D., Pai R.L., Srkalovic M.B., Tamakloe M.A., Rasheed R., Ziglar J., Khor J., Parente S.A.T. (2020). Increased mTOR activation in idiopathic multicentric Castleman disease. Blood.

[43] Kadoba K., Waki D., Nishimura K., Mukoyama H., Saito R., Murabe H., Yokota T. (2020). Development of severe thrombocytopenia with TAFRO syndrome-like features in a patient with rheumatoid arthritis treated with a Janus kinase inhibitor: A case report. Medicine.

[44] Wakiya R., Kameda T., Takeuchi Y., Ozaki H., Nakashima S., Shimada H., Kadowaki N., Dobashi H. (2021). Sequential change in serum VEGF levels in a case of tocilizumab-resistant TAFRO syndrome treated effectively with rituximab. Mod. Rheumatol. Case Rep..

[45] Kikuchi T., Shimizu T., Toyama T., Abe R., Okamoto S. (2017). Successful treatment of TAFRO syndrome with tocilizumab, prednisone, and cyclophosphamide. Intern. Med..

[46] Williams C., Phillips A., Aggarwal V., Slonim L.B., Fajgenbaum D.C., Karmali R. (2021). TAFRO syndrome and elusive diagnosis of idiopathic multicentric Castleman disease treated with empiric anti-interleukin-6 therapy. Case Rep. Oncol..

[47] Kakutani T., Nunokawa T., Chinen N., Tamai Y. (2022). Treatment-resistant idiopathic multicentric Castleman disease with thrombocytopenia, anasarca, fever, reticulin fibrosis, renal dysfunction, and organomegaly managed with Janus kinase inhibitors: A case report. Medicine.

[48] Killian M., Viel S., Chalayer E., Forest F., Grange S., Bonnefoy P.B., Oksenhendler E., Cathébras P., Paul S. (2021). JAK1/2 inhibition in severe TAFRO syndrome: A case report. Ann. Intern. Med..

[49] van Rhee F., Wong R.S., Munshi N., Rossi J.F., Ke X.Y., Fosså A., Simpson D., Capra M., Liu T., Hsieh R.K. (2014). Siltuximab for multicentric Castleman’s disease: A randomised, double-blind, placebo-controlled trial. Lancet Oncol..

[50] Igawa T., Sato Y. (2018). TAFRO syndrome. Hematol. Oncol. Clin. N. Am..

[51] Lust H., Gong S., Remiker A., Rossoff J. (2021). Idiopathic multicentric Castleman disease with TAFRO clinical subtype responsive to IL-6/JAK inhibition: A pediatric case series. Pediatr. Blood Cancer.

[52] Liu X.R., Tian M. (2022). Glucocorticoids combined with tofacitinib in the treatment of Castleman’s disease: A case report. World J. Clin. Cases.

[53] Sakashita K., Murata K., Takamori M. (2018). TAFRO syndrome: Current perspectives. J. Blood Med..

[54] Ocio E.M., Sanchez-Guijo F.M., Diez-Campelo M., Castilla C., Blanco O.J., Caballero D., San Miguel J.F. (2005). Efficacy of rituximab in an aggressive form of multicentric Castleman disease associated with immune phenomena. Am. J. Hematol..

[55] Watanabe M., Haji Y., Hozumi M., Amari Y., Mizuno Y., Ito T., Kato M., Okada M. (2023). Combined B-cell immunomodulation with rituximab and Belimumab in severe, refractory TAFRO syndrome associated with Sjögren’s syndrome: A case report. Mod. Rheumatol. Case Rep..

[56] Dufour J.H., Dziejman M., Liu M.T., Leung J.H., Lane T.E., Luster A.D. (2002). IFN-γ-inducible protein 10 (IP-10; CXCL10)-deficient mice reveal a role for IP-10 in effector T cell generation and trafficking. J. Immunol..

[57] Chronowski G.M., Ha C.S., Wilder R.B., Cabanillas F., Manning J., Cox J.D. (2001). Treatment of unicentric and multicentric Castleman disease and the role of radiotherapy. Cancer.

[58] Nishi J., Arimura K., Utsunomiya A., Yonezawa S., Kawakami K., Maeno N., Ijichi O., Ikarimoto N., Nakata M., Kitajima I. (1999). Expression of vascular endothelial growth factor in sera and lymph nodes of the plasma cell type of Castleman’s disease. Br. J. Haematol..

[59] Stary G.N., Kohrgruber N., Herneth A.M., Gaiger A., Stingl G., Rieger A. (2008). Complete regression of HIV-associated multicentric castleman disease treated with rituximab and thalidomide. Aids.

[60] Miltenyi Z., Toth J., Gonda A., Tar I., Remenyik E., Illes A. (2009). Successful immunomodulatory therapy in castleman disease with paraneoplastic pemphigus vulgaris. Pathol. Oncol. Res..

[61] Jung C.P., Emmerich B., Goebel F.D., Bogner J.R. (2004). Successful treatment of a patient with HIV-associated multicentric castleman disease (MCD) with thalidomide. Amm. J. Hematol..

[62] Tatekawa S., Umemura K., Fukuyama R., Kohno A., Taniwaki M., Kuroda J., Morishita Y. (2015). Thalidomide for tocilizumab-resistant ascites with TAFRO syndrome. Clin. Case Rep..

[63] Chen T., Feng C., Zhang X., Zhou J. (2023). TAFRO syndrome: A disease that known is half cured. Hematol. Oncol..

[64] Caballero J.C., Conejero N., Solan L., de la Pinta F.J.D., Cordoba R., Lopez-Garcia A. (2024). Unraveling TAFRO syndrome: An In-Depth Look at the Pathophysiology, Management, and Future Perspectives. Biomedicines.

